# Temporal Patterns of Serum Neuroprotectin D1 and Resolvin D1 After Motor and Nonmotor Epileptic Seizures: A Prospective Repeated-Measures Study

**DOI:** 10.3390/diagnostics16213706

**Published:** 2026-11-09

**Authors:** Ahmet Sengul, Irem Tasci, Ayse Berilgen Gurgoze, Sait Albayrak, Selda Telo

**Affiliations:** 1 Department of Neurology, Faculty of Medicine, Fırat University, 23119 Elazığ, Türkiye; asengul@firat.edu.tr (A.S.); aberilgen@firat.edu.tr (A.B.G.); saitakademik@outlook.com (S.A.); 2 Department of Biochemistry, Faculty of Medicine, Fırat University, 23119 Elazığ, Türkiye; drseldatelo@hotmail.com

**Keywords:** epilepsy, neuroprotectin D1, resolvin D1, neuroinflammation, postictal period, specialized pro-resolving mediators

## Abstract

**Highlights:**

- NP-1 increased between the early postictal and seizure-free measurements (*p* < 0.001; r = 0.770).
- RvD1 did not change significantly between patient sampling periods (*p* = 0.139).
- During seizure freedom: the relative RvD1 deficit exceeded the NP-1 deficit (*p* = 0.001; r = 0.462).
- Different individual significance results do not establish different temporal trajectories.

**Abstract:**

**Objective**: Neuroinflammatory activation is well recognized in epilepsy, whereas the temporal behavior of specialized pro-resolving mediator (SPM) responses after seizures remains poorly defined. We evaluated changes in serum neuroprotectin D1 (NP-1) and resolvin D1 (RvD1) between the early postictal period and a seizure-free period of at least 1 month and compared both time points with healthy controls. **Methods**: This prospective controlled repeated-measures study included 48 adults with epilepsy and 50 healthy controls. Blood was obtained within 3 h after a seizure and again after at least 1 month of seizure freedom; controls provided one sample. Serum NP-1 and resolvin D1 (RvD1) were measured using commercial enzyme-linked immunosorbent assay (ELISA) kits. The prespecified primary analysis compared paired postictal and seizure-free measurements using the Wilcoxon signed-rank test. Patient-control comparisons were considered secondary/exploratory. **Results**: Median NP-1 increased from 50.01 ng/mL (IQR 3.58–98.62) postictally to 67.78 ng/mL (59.81–121.81) during the seizure-free period (Z = −5.333, *p* < 0.001, r = 0.770). RvD1 did not change significantly (326.67 [207.77–899.95] vs. 441.47 [223.67–710.81] pg/mL; *p* = 0.139, r = 0.215). Both mediators were lower than controls postictally (both *p* < 0.001). During seizure freedom, NP-1 and RvD1 remained lower than controls (*p* = 0.008 and *p* < 0.001, respectively). The absolute percentage difference from the control median was greater for RvD1 than for NP-1 during seizure freedom (60.74% vs. 42.84%; *p* = 0.001, r = 0.462). **Conclusions**: The pro-resolving response in epilepsy appears temporally heterogeneous. NP-1 showed substantial within-patient recovery, whereas Resolvin D1 remained persistently reduced. These findings are hypothesis-generating and support larger serial-sampling studies with targeted lipidomic validation rather than establishing either mediator as a diagnostic biomarker.

## 1. Introduction

Epilepsy is not merely a disorder of recurrent seizures but a dynamic network disease in which neuronal hyperexcitability interacts with glial activation, blood–brain barrier dysfunction, and immune signaling [1,2]. Neuroinflammation is increasingly regarded as an active pathophysiologic component that can influence epileptogenesis and seizure recurrence rather than simply a consequence of seizures [1]. Accordingly, understanding epilepsy requires examination not only of how strongly inflammatory pathways are activated but also of how efficiently inflammation is terminated.

Resolution of inflammation is an active biological program regulated by specialized pro-resolving mediators (SPMs), including resolvins, protectins, maresins, and lipoxins [3,4]. These mediators can restrain proinflammatory signaling, promote clearance of cellular debris, and support restoration of tissue homeostasis. In central nervous system disorders, inadequate activation of these pathways may contribute to persistence of inflammation and secondary neuronal injury [4].

Docosahexaenoic acid-derived neuroprotectin D1 (NP-1) and resolvin D1 (RvD1) are of particular interest because of their neuroprotective and pro-resolving actions. In an experimental model of epileptogenesis, protectin D1 reduced proinflammatory cytokine expression and suppressed epileptogenesis [5,6]. More recently, circulating and cerebrospinal fluid concentrations of RvD1 and Resolvin E1 were reported to be reduced in patients with chronic epilepsy [7]. However, human data describing how different SPM pathways behave within the same individual immediately after a seizure and again during a seizure-free period remain limited.

We therefore examined serum NP-1 and RvD1 at two clinically distinct time points. Our primary hypothesis was that the two mediators would not show identical temporal behavior between the early postictal and seizure-free periods. We compared paired measurements obtained within 3 h after a seizure and after at least 1 month of seizure freedom; healthy-control comparisons were treated as secondary analyses providing biological context for the longitudinal findings.

## 2. Methods

### 2.1. Study Design and Participants

This prospective controlled clinical study was conducted in the Department of Neurology, Faculty of Medicine, Fırat University. Adults aged 18 years or older who presented to the emergency department, neurology inpatient service, or neurology outpatient clinic after an epileptic seizure and met the International League Against Epilepsy clinical definition of epilepsy were evaluated [8]. The study was approved by the Fırat University Non-Interventional Research Ethics Committee (10 October 2024; decision no. 2024/13-49), and written informed consent was obtained from all participants.

Exclusion criteria were an additional neurologic disorder other than epilepsy, active infection, systemic inflammatory or autoimmune disease, malignancy, severe hepatic or renal failure, pregnancy, or inability to provide informed consent. Healthy controls had no history of epilepsy, active neurologic disease, or systemic inflammatory disease. Using G*Power 3.1 with alpha = 0.05, power = 0.95, and an assumed effect size of 0.50, the minimum sample size for the repeated-measures comparison was 47 patients [9]. The final study included 48 patients and 50 controls.

### 2.2. Sampling and Laboratory Analysis

The first venous blood sample from each patient was obtained within 3 h after the index seizure (early postictal period), and the second was obtained after at least 1 month of seizure freedom (seizure-free period). Healthy controls provided a single sample. Age, sex, seizure semiology, antiseizure medication (ASM) use, and number of ASMs were recorded. The recorded seizure categories included generalized motor *(n* = 29), focal motor (*n* = 9), generalized nonmotor (*n* = 7), and focal nonmotor (*n* = 3) events. 

Approximately 2 mL of venous blood was collected into biochemistry tubes and centrifuged at 3000× *g* for 10 min. The resulting serum was stored at −80 °C until analysis. Repeated freeze–thaw cycles were avoided. Serum NP-1 and RvD1 were measured using commercial sandwich enzyme-linked immunosorbent assay (ELISA) kits (YL Biont, Shanghai, China; catalogue numbers YLA4728HU and YLA4193HU, respectively), following the manufacturers’ procedures. Absorbance was measured at 450 nm using a BioTek Epoch 2 microplate reader (Agilent Technologies, Winooski, VT, USA). The manufacturer-reported NP-1 detection range was 0.5–150 ng/mL, with analytical sensitivity of 0.26 ng/mL. The reported RvD1 analytical sensitivity was 4.12 pg/mL. For both kits, intra-assay coefficients of variation were <8% and inter-assay coefficients of variation were <10%. NP-1 concentrations are reported in ng/mL and RvD1 concentrations in pg/mL.

The manufacturer-specified assay ranges were 0.5–150 ng/mL for NP-1 and 10–1000 pg/mL for RvD1, with reported analytical sensitivities of 0.26 ng/mL and 4.12 pg/mL, respectively. For both kits, the manufacturer-reported intra-assay and inter-assay coefficients of variation were <8% and <10%, respectively. Serum samples from patients and healthy controls were analyzed in two separate runs, with identical results obtained in both runs. These assay ranges describe analytical performance rather than population-based biological reference intervals. The 50 healthy volunteers served as a study-specific comparison group, and the corresponding control medians were used to calculate relative percentage differences. This study was not designed to establish population-based biological reference intervals. NP-1 and RvD1 concentrations are reported in ng/mL and pg/mL, respectively.

### 2.3. Statistical Analysis

Analyses were performed using IBM SPSS Statistics version 26.0 and Python 3.11. Distributional assumptions were evaluated with the Shapiro–Wilk test and graphical inspection. Because NP-1, RvD1, and age were non-normally distributed, continuous variables are presented primarily as median and interquartile range (IQR).

The primary analysis compared early postictal (EN) and seizure-free (ER) measurements within the same 48 patients using the Wilcoxon signed-rank test. Healthy controls contributed one independent observation each; comparisons of controls with EN and with ER used separate Mann–Whitney U tests and were secondary/exploratory. EN and ER were not analyzed as independent groups in a three-group omnibus test. Kruskal–Wallis tests were used solely for comparisons between independent seizure semiology subgroups within each time period. Medication-count subgroups were compared using Mann–Whitney U tests within each time point. Effect size r was calculated as |Z|/√N, using the number of paired observations for paired tests and the total number of participants in the two groups for independent comparisons. For each patient and mediator, the percentage difference from the control median was calculated as [(patient value − control median)/control median] × 100; the absolute percentage differences for the two mediators were compared within patients using the paired Wilcoxon test. These comparisons describe relative distance from a shared control reference and do not directly test a mediator-by-time interaction. Tests were two-sided. Reported *p*-values from the retained analyses are unadjusted, and *p* < 0.05 was the nominal significance threshold; secondary analyses are exploratory.

## 3. Results

### 3.1. Participant Characteristics

First, a cohort was defined to provide context for paired analyses and subgroup analyses (Table 1).

The study included 48 patients with epilepsy (30 women [62.5%] and 18 men [37.5%]). Median age was 38 years (IQR: 31–50; range: 20–87). Most patients were receiving antiseizure treatment (91.7%; *n* = 44), and levetiracetam was the most commonly used agent (58.3%). Eighteen patients (37.5%) were receiving monotherapy and 26 (54.1%) polytherapy (≥2 drugs). Generalized motor seizures were the most common seizure type (60.4%; *n* = 29), followed by focal motor (18.8%), generalized nonmotor (14.6%), and focal nonmotor (6.2%) seizures. These characteristics are summarized in Table 1.

### 3.2. Paired Changes Between Sampling Periods

**Figure 1:**
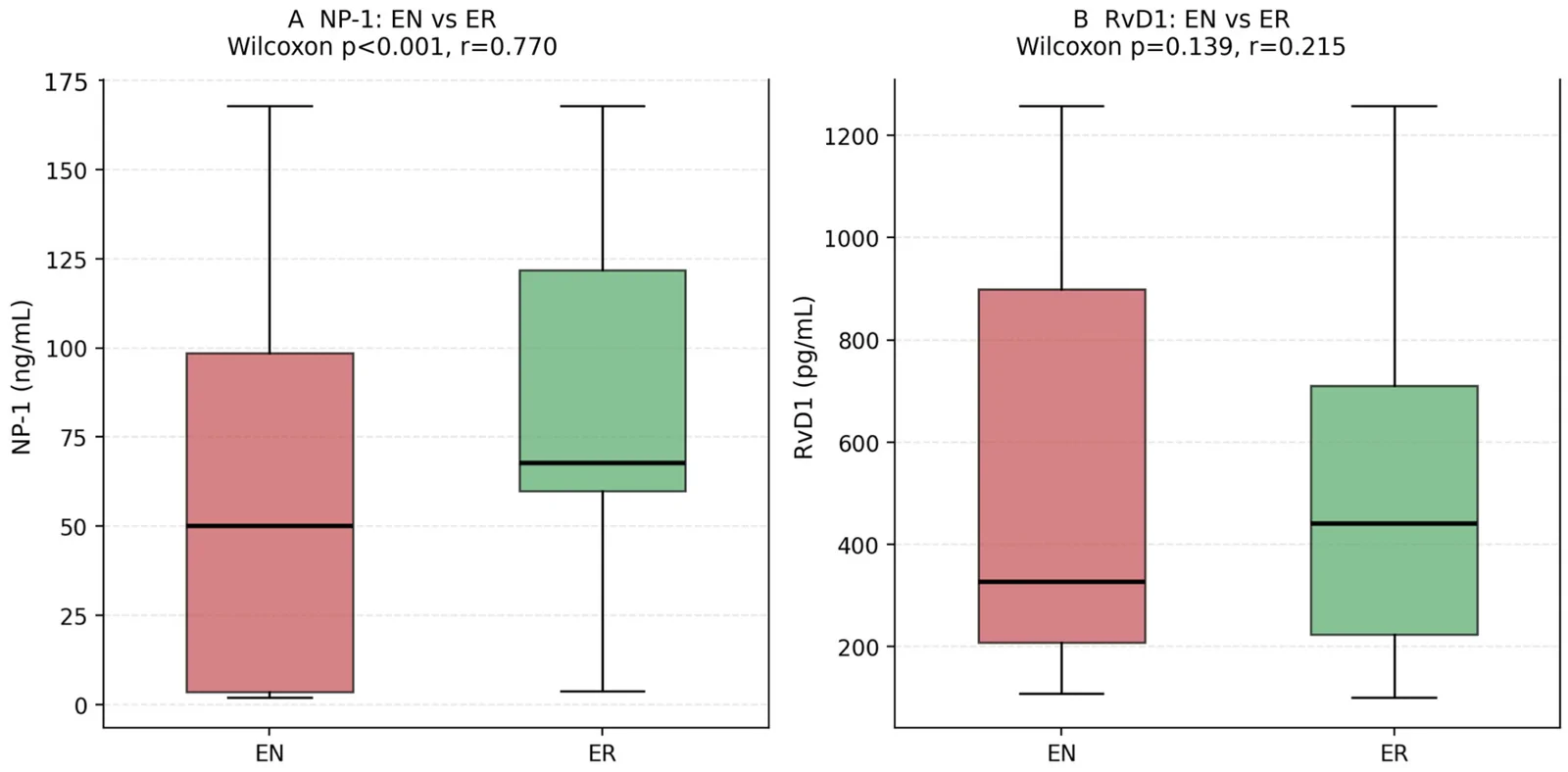
Serum NP-1 (**A**) and RvD1 (**B**) during EN and ER in 48 patients. Colored boxes show the interquartile ranges and horizontal lines the reported medians; these are not individual patient trajectories. Panel headings identify the paired Wilcoxon comparisons: NP-1, *p* < 0.001; RvD1, *p* = 0.139. EN: early postictal period (within 3 h); ER: seizure-free period (at least 1 month); NP-1: neuroprotectin D1; RvD1: resolvin D1.

To evaluate the changes between EN and ER, measurements from the same patients were compared (Table 2 and Figure 1).

NP-1 was higher during ER than EN (67.78 vs. 50.01 ng/mL; Z = −5.333, *p* < 0.001, r = 0.770). RvD1 did not differ significantly between the two measurements (441.47 vs. 326.67 pg/mL; Z = −1.487, *p* = 0.139, r = 0.215).

**Figure 2:**
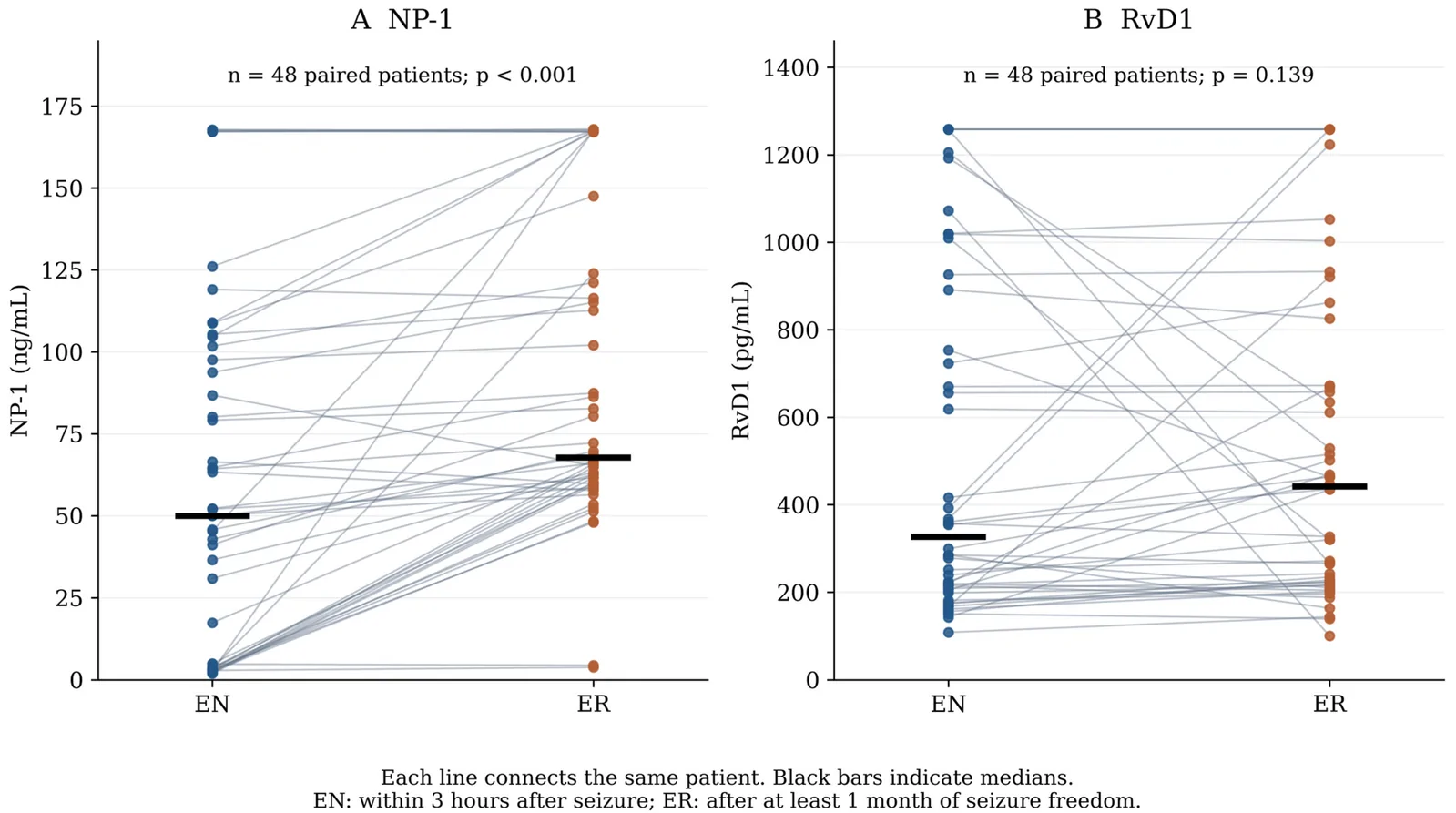
Individual paired serum NP-1 (**A**) and RvD1 (**B**) measurements during EN and ER in 48 patients. Each line connects the two measurements from the same patient; blue and orange points indicate EN and ER, respectively. Black horizontal bars indicate medians. Two-sided paired Wilcoxon signed-rank tests: NP-1, *p* < 0.001; RvD1, *p* = 0.139. EN: early postictal period, within 3 h after the index seizure; ER: seizure-free period, after at least 1 month without seizures; NP-1: neuroprotectin D1 (ng/mL); RvD1: resolvin D1 (pg/mL).

Individual EN–ER measurements are shown in Figure 2. NP-1 increased in 40 patients and decreased in 8; RvD1 increased in 30 patients and decreased in 18. No unchanged values were observed for either marker.

### 3.3. Comparisons with Healthy Controls

To place the paired measurements into context, each patient time point was compared with healthy controls (Table 3 and Table 4).

Both markers were lower during EN than in controls (Table 3). Median NP-1 was 50.01 vs. 118.02 ng/mL (U = 1849, Z = 4.612, *p* < 0.001, r = 0.466), and median RvD1 was 326.67 vs. 1124.37 pg/mL (U = 1875, Z = 4.797, *p* < 0.001, r = 0.485).

**Figure 3:**
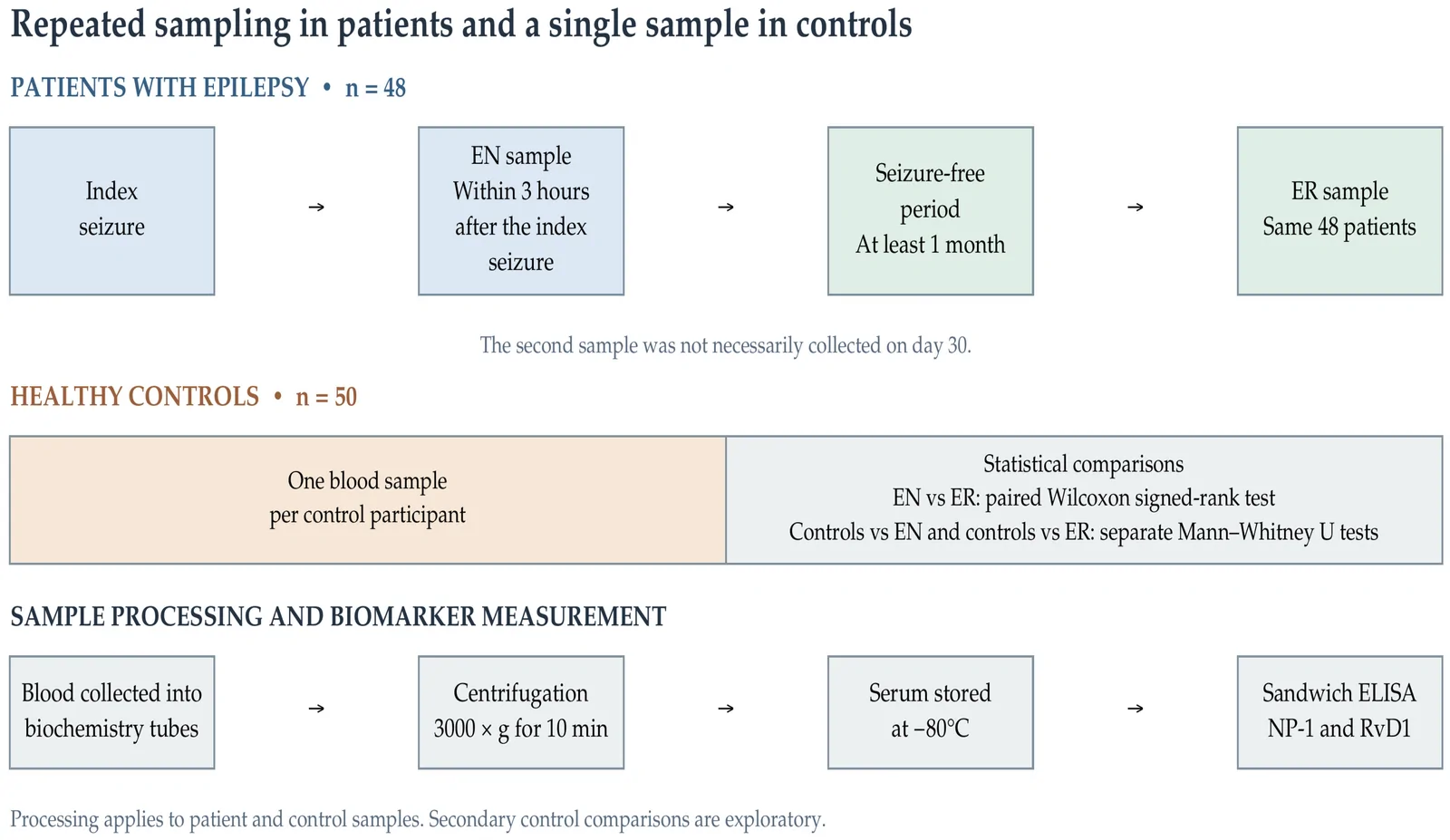
Study sampling timeline, specimen processing, and main statistical comparisons. The same 48 patients provided an early postictal sample within 3 h after the index seizure (EN) and a second sample after at least 1 month of seizure freedom (ER). Fifty healthy controls provided one sample each. Blood samples were centrifuged at 3000× *g* for 10 min, and serum was stored at −80 °C until measurement of neuroprotectin D1 (NP-1) and resolvin D1 (RvD1) using sandwich enzyme-linked immunosorbent assays (ELISAs). EN–ER measurements were compared using the paired Wilcoxon signed-rank test. Separate comparisons of controls with each patient time point used the Mann–Whitney U test and were exploratory.

The sampling timeline, serum processing procedures, biomarker measurements, and statistical comparisons are summarized in Figure 3.

During ER, NP-1 remained lower than in controls (67.78 vs. 118.02 ng/mL; U = 1576, Z = 2.672, *p* = 0.008, r = 0.270; Table 4). RvD1 also remained lower than in controls (441.47 vs. 1124.37 pg/mL; U = 1858, Z = 4.680, *p* < 0.001, r = 0.473). These are separate independent-group comparisons, not a three-group repeated-measures analysis.

### 3.4. Relative Differences from Control Medians 3

**Figure 4:**
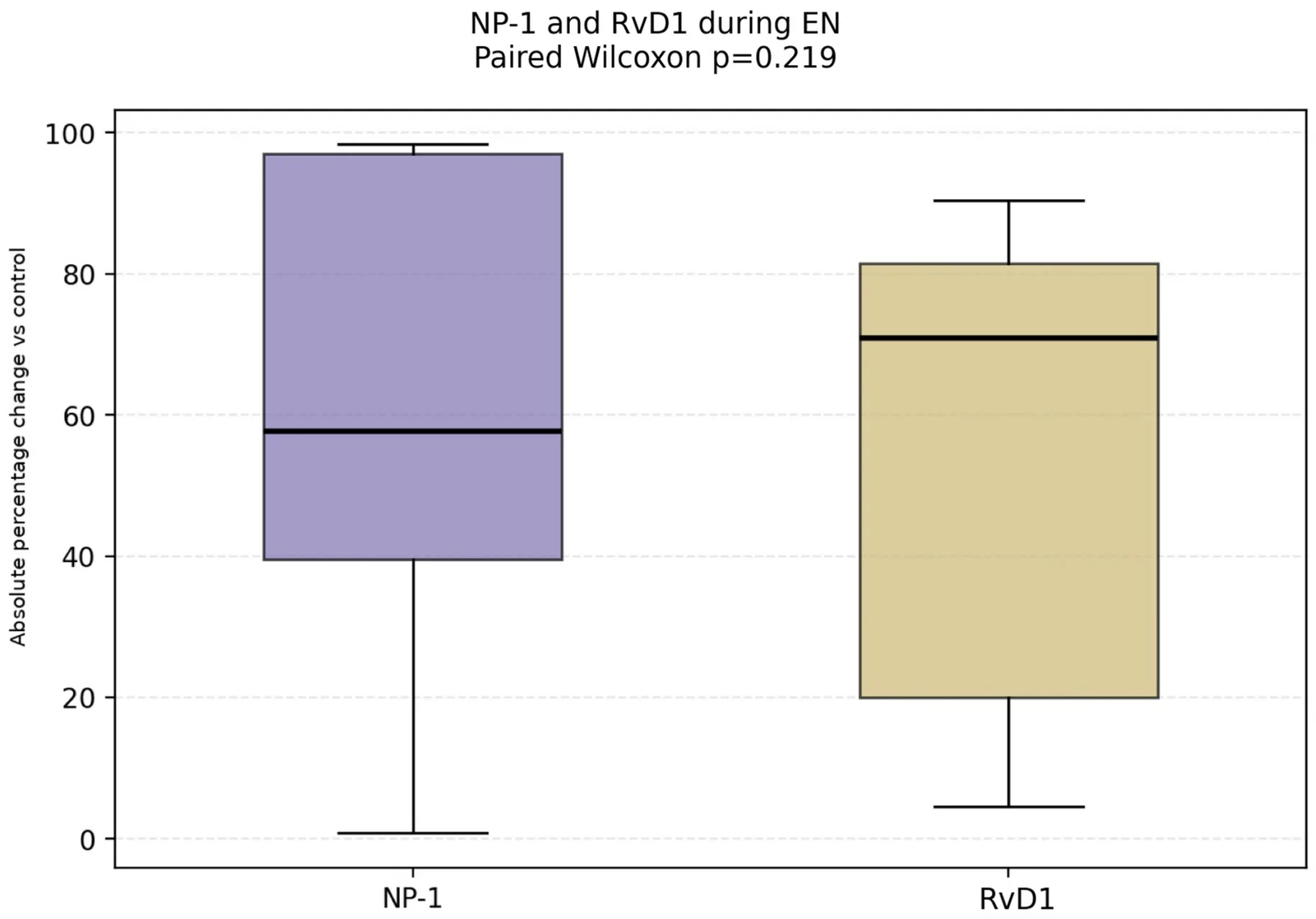
Absolute percentage differences from the control medians during EN. Colored boxes show interquartile ranges and horizontal lines show medians. Paired Wilcoxon test, *p* = 0.219. EN: early postictal period (within 3 h); NP-1: neuroprotectin D1; RvD1: resolvin D1; |%Δ|: absolute percentage difference from the corresponding control median.

**Figure 5:**
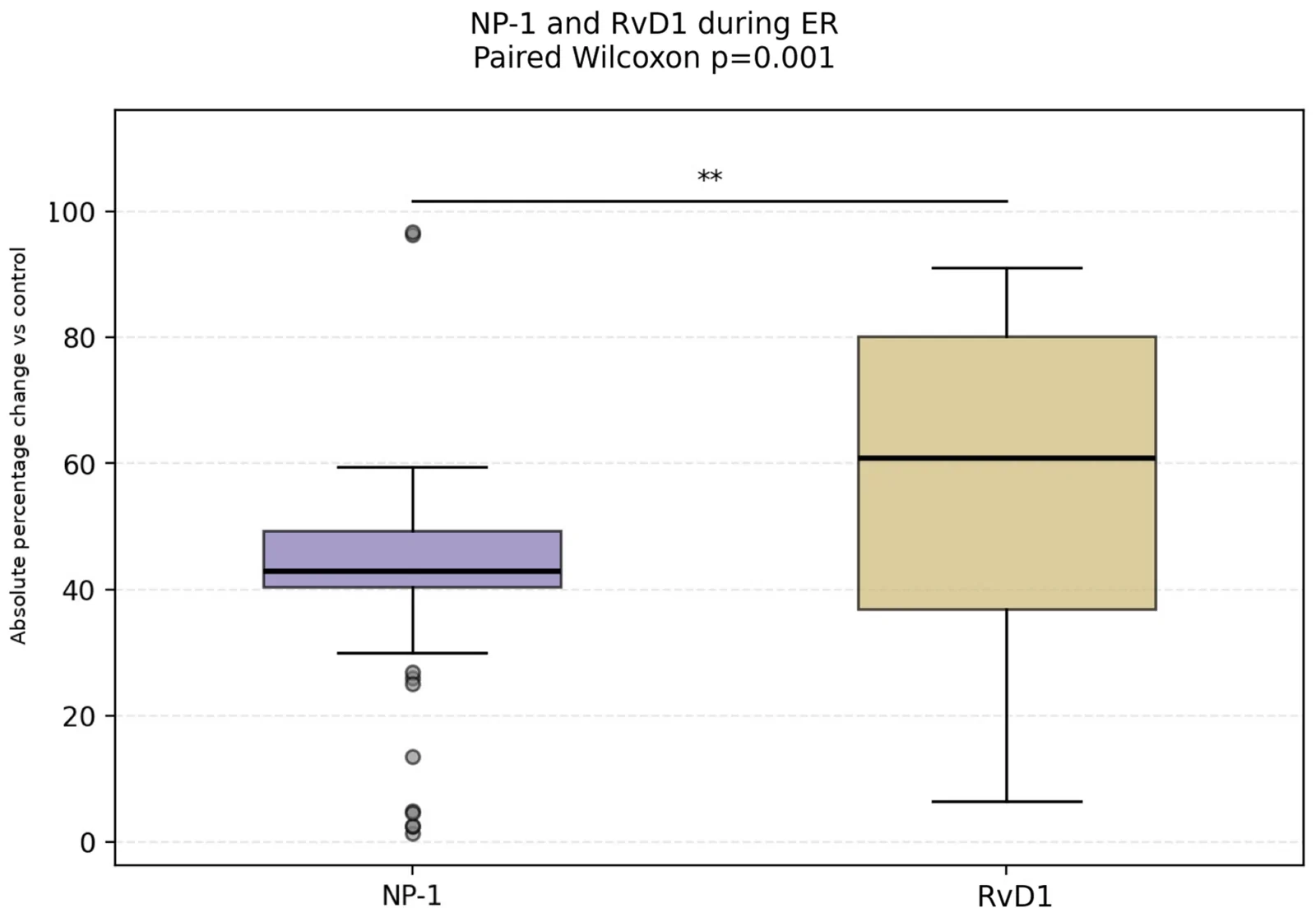
Absolute percentage differences from the control medians during ER. Colored boxes show interquartile ranges and horizontal lines show medians. ** indicates a significant difference between NP-1 and RvD1 (paired Wilcoxon signed-rank test, *p* = 0.001). Paired Wilcoxon test, *p* = 0.001. ER: seizure-free period (at least 1 month); NP-1: neuroprotectin D1; RvD1: resolvin D1; |%Δ|: absolute percentage difference from the corresponding control median.

The two mediators were compared on a common percentage scale, using the control median for each marker as a reference. Absolute percentage differences were compared within patients using the paired Wilcoxon test (Table 5 and Table 6; Figure 4 and Figure 5).

During EN, median signed percentage differences were −57.63% for NP-1 and −70.95% for RvD1. Median absolute percentage differences were 57.63% and 70.95%, respectively; their paired comparison was not statistically significant (T = 467, Z = 1.241, *p* = 0.219, r = 0.179; Table 5 and Figure 4).

During ER, median signed percentage differences were −42.57% for NP-1 and −60.74% for RvD1. Median absolute percentage differences were 42.84% and 60.74%, respectively; the paired comparison was significant (T = 276, Z = −3.200, *p* = 0.001, r = 0.462; Table 6 and Figure 5).

### 3.5. Exploratory Comparisons by Seizure Semiology

**Figure 6:**
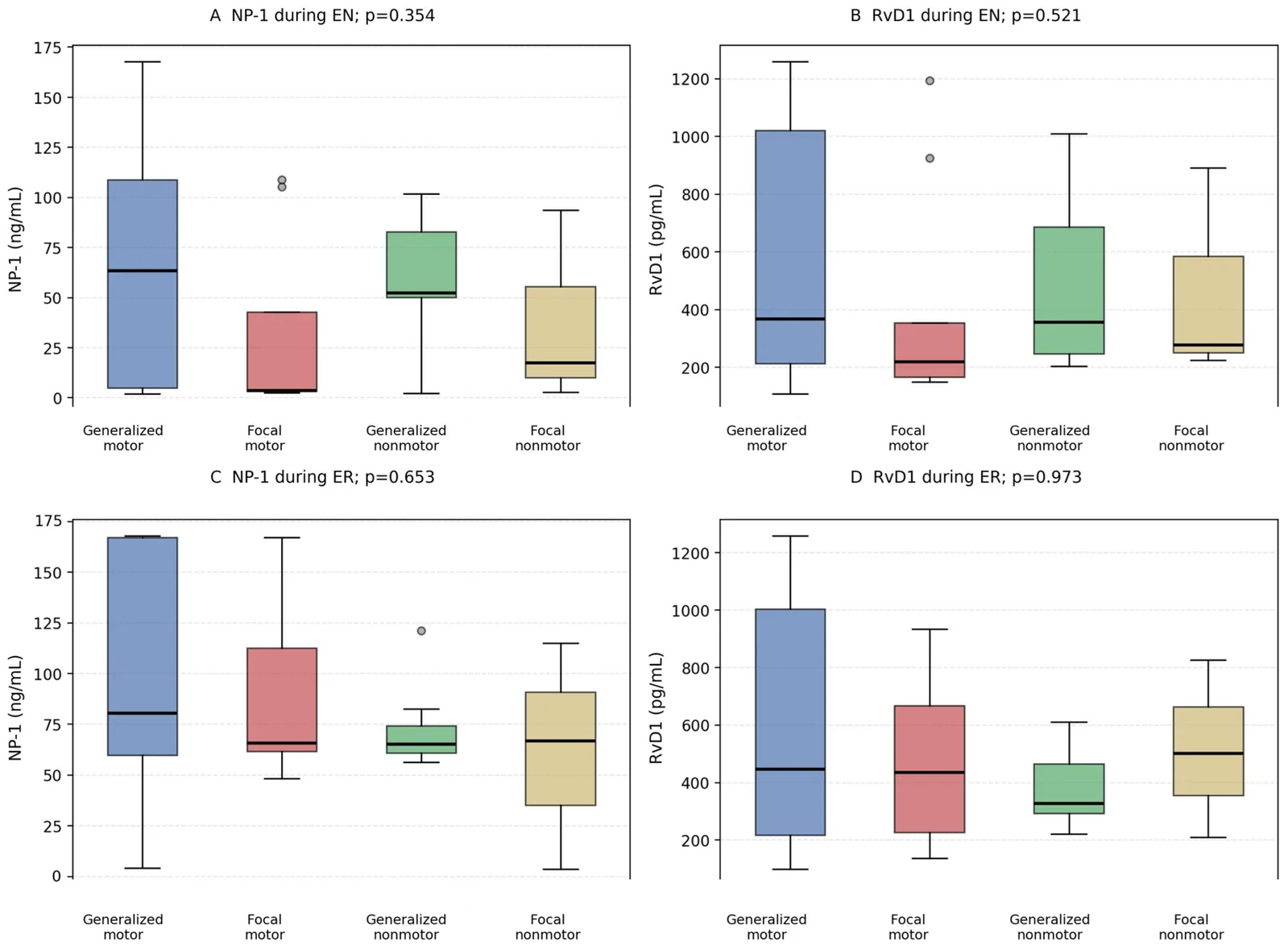
Marker concentrations by seizure semiology. (**A**) NP-1 during EN; (**B**) RvD1 during EN; (**C**) NP-1 during ER; (**D**) RvD1 during ER. Colored boxes show interquartile ranges and horizontal lines show medians from Table 7. The individual dots represent outliers, defined as observations falling more than 1.5 times the interquartile range (IQR) below the first quartile (Q1) or above the third quartile (Q3). These points indicate individual values outside the expected distribution range and were retained in the analysis. Generalized motor, *n* = 29; focal motor, *n* = 9; generalized nonmotor, *n* = 7; focal nonmotor, *n* = 3. EN: early postictal period (within 3 h); ER: seizure-free period (at least 1 month); NP-1: neuroprotectin D1; RvD1: resolvin D1.

Within each sampling period, it was assessed whether marker levels differed across four independent subgroups defined by seizure semiology (Table 7 and Figure 6).

Neither marker differed significantly across seizure-semiology subgroups during EN or ER (all *p* > 0.05; Table 7). The generalized motor subgroup included 29 patients, compared with 9 focal motor, 7 generalized nonmotor, and 3 focal nonmotor patients.

### 3.6. Exploratory Comparisons by Medication Count

**Figure 7:**
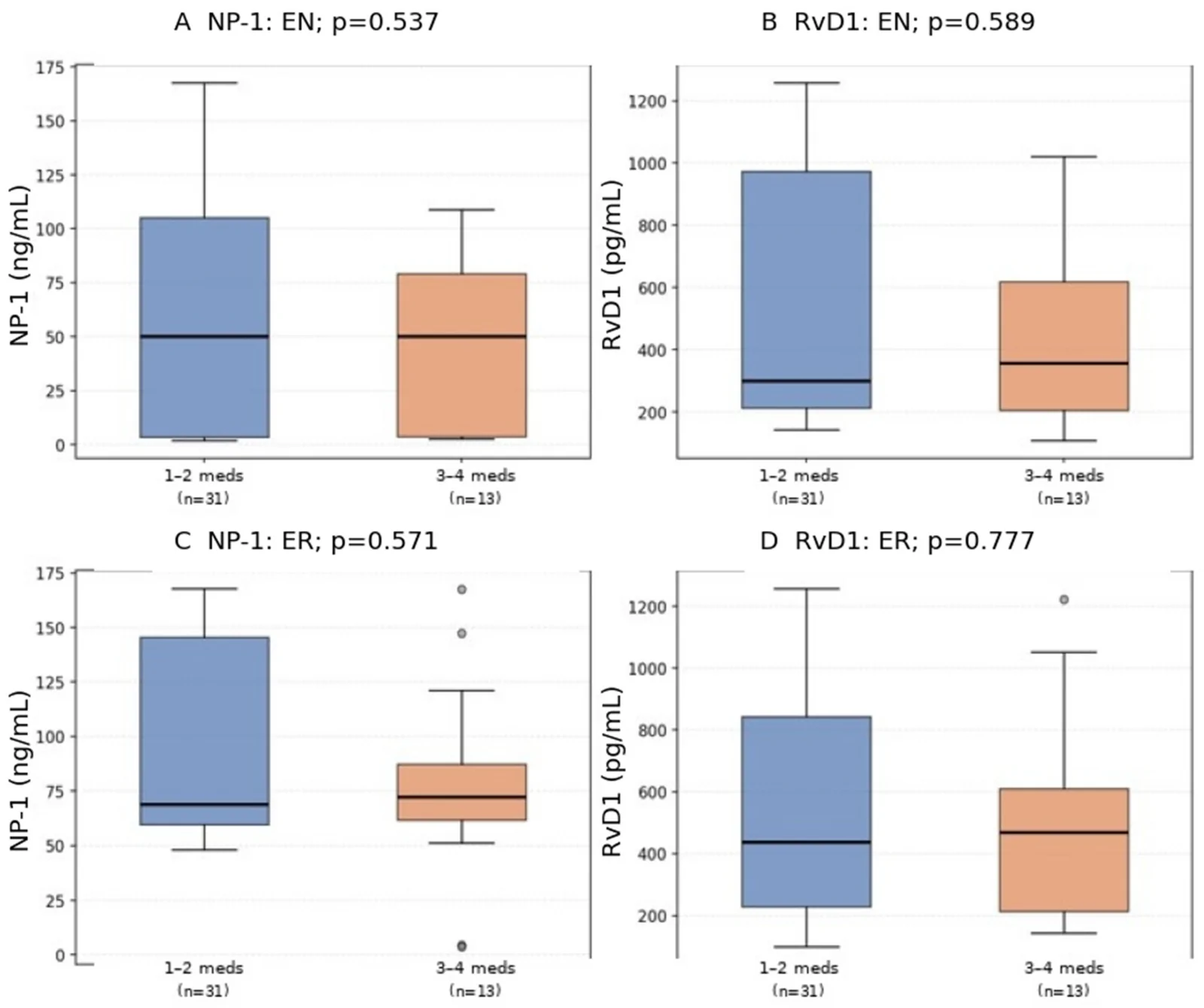
Marker concentrations by ASM count (1–2 vs. 3–4 medications). (**A**,**B**) EN; (**C**,**D**) ER. Colored boxes show interquartile ranges and horizontal lines show medians. ASM: antiseizure medication; EN: early postictal period (within 3 h); ER: seizure-free period (at least 1 month); NP-1: neuroprotectin D1; RvD1: resolvin D1.

We assessed associations with medication count by comparing patients taking 1–2 ASMs (*n* = 31) with those taking 3–4 ASMs (*n* = 13), separately during EN and ER (Table 8 and Figure 7). Four untreated patients were excluded.

Neither marker differed significantly by medication count during EN or ER (all *p* > 0.05; Table 8 and Figure 7). During EN, median NP-1 was 50.05 ng/mL with 1–2 medications and 49.97 ng/mL with 3–4 medications (*p* = 0.537); median RvD1 was 299.31 and 356.74 pg/mL, respectively (*p* = 0.589). During ER, *p*-values were 0.571 for NP-1 and 0.777 for RvD1. Reported effect sizes were r < 0.10.

## 4. Discussion

Epilepsy is increasingly understood not merely as a chronic neurological disorder characterized by recurrent seizures, but as a dynamic disease process shaped by neuronal hyperexcitability, glial activation, disruption of blood–brain barrier integrity, and immune-mediated signaling networks [1]. During epileptogenesis, the inflammatory response is increasingly recognized not only as a consequence of epileptic seizures but also as an active pathophysiological component that contributes to the formation and maintenance of epileptic networks and to seizure recurrence [1]. In the central nervous system, microglial and astrocytic activation increases proinflammatory cytokines and chemokines, including IL-1β, IL-6, and TNF-α. These changes have been linked to mechanisms that may facilitate neuronal hyperexcitability by enhancing glutamatergic transmission and NMDA receptor–mediated excitability [1]. Accordingly, neuroinflammation in epilepsy should be regarded not simply as an accompanying histopathological or biochemical finding, but as a functional process that may influence seizure threshold, disease severity, and progression to chronic epilepsy [1].

The conventional framework for explaining the relationship between neuroinflammation and epilepsy has largely focused on increased levels of proinflammatory mediators. In recent years, however, this framework has expanded to include the active resolution of inflammation and the restoration of tissue homeostasis [4]. Resolution is not the passive waning of an inflammatory response; it is a programmed, energy-dependent regulatory process governed by biologically active lipid mediators known as specialized pro-resolving mediators (SPMs) [4]. These mediators include resolvins, protectins, maresins, and lipoxins. They limit neutrophil infiltration, enhance macrophage-mediated efferocytosis, suppress proinflammatory cytokine production, reduce oxidative stress, and support tissue repair [4]. In central nervous system disorders, impaired SPM activity may contribute to a failure to resolve inflammation and the persistence of a chronic neuroinflammatory environment [10].

The concept of inflammatory resolution, or pro-resolving capacity, is particularly relevant to epilepsy. Recurrent seizures can trigger acute inflammatory fluctuations, while the underlying epileptic condition may also be characterized by persistent, low-grade inflammatory activation [1]. It is therefore necessary to distinguish biomarkers that change transiently after a seizure from more stable biomarkers that reflect the chronic epileptic condition [7]. In a clinical study of patients with epilepsy that examined the chronic disease state, RvD1 and Resolvin E1 levels were lower in both plasma and cerebrospinal fluid. This finding suggests that epilepsy may involve not only activation of inflammation but also insufficient pro-resolving activity [7]. In the same study, Resolvin E1 levels showed an independent inverse association with seizure severity, suggesting that a deficiency in pro-resolving mediators may be related to clinical disease burden [7]. In this context, biomarker assessment in epilepsy should consider reduced pro-resolving capacity alongside elevated levels of proinflammatory molecules [7].

The findings of this study show that neuroprotectin D1 (NP-1) and RvD1 levels were markedly lower in patients with epilepsy during the early postictal period than in the control group (Table 3). This finding suggests that, alongside inflammatory activation, the response of lipid mediators involved in the resolution of inflammation may be substantially attenuated after a seizure. NP-1 levels were lower within the first 3 h after a seizure than after 1 month of seizure freedom, suggesting that this molecule may serve as a state-dependent biomarker sensitive to acute seizure burden. In contrast, RvD1 levels remained lower than those in the control group both within the first 3 h after a seizure and after 1 month of seizure freedom. This pattern suggests that disruption of the RvD1 pathway may be a more persistent pathophysiological feature that cannot be explained solely by the acute effects of a seizure. Together, these distinct patterns suggest that specialized pro-resolving mediators (SPMs) do not behave uniformly in epilepsy: protectins and resolvins may reflect different biological aspects of the disease and may contribute to its pathogenesis.

The marked reduction in NP-1 levels within the first 3 h after a seizure, followed by partial recovery during the seizure-free period, is relevant to the temporal dynamics of protectin-family specialized pro-resolving mediators (SPMs) in the acute neuroinflammatory response (Table 2; Figure 1 and Figure 2). Protectins are lipid mediators derived from omega-3 fatty acid metabolism that contribute to the resolution of neuroinflammation [4]. In an experimental model of epileptogenesis, administration of n-3 DPA-derived protectin D1 reduced IL-1β and TNF-α expression [5]. This suggests that protectin D1 may do more than reflect inflammation; it may also modulate inflammatory pathways [5]. In the same study, protectin D1 treatment approximately halved spontaneous seizure frequency, shortened seizure duration, and substantially reduced total seizure time, supporting the potential biological effects of SPMs on epileptic network activity [5]. In light of these findings, the early postictal reduction in NP-1 observed in the present study may indicate a transient insufficiency of protectin-mediated anti-inflammatory responses following ictal epileptic activity.

In this study, NP-1 levels were significantly lower within the first 3 h after an epileptic seizure than after 1 month of seizure freedom. This finding suggests that the inflammatory burden immediately following a seizure may suppress protectin-family specialized pro-resolving mediators (SPMs). The increase in NP-1 levels after 1 month of seizure freedom suggests that the protectin-mediated anti-inflammatory response is not permanently lost and may partially recover during a seizure-free period (Table 2; Figure 1 and Figure 2). However, NP-1 levels did not fully approach those of the control group after 1 month of seizure freedom (Table 4), suggesting that baseline SPM capacity may remain impaired despite partial recovery from the acute postictal response. These findings support further evaluation of NP-1 as a potential longitudinal marker of the early postictal response and its subsequent partial recovery (Table 2; Figure 1 and Figure 2). Moreover, the significantly lower serum NP-1 levels observed within the first 3 h after a seizure, compared with healthy controls, raise the possibility that NP-1 could be investigated as a biomarker for distinguishing epileptic seizures from psychogenic nonepileptic seizures, analogous to the proposed clinical use of prolactin.

Resolvins are potent endogenous mediators of inflammatory resolution derived from the metabolism of docosahexaenoic acid and eicosapentaenoic acid [6]. RvD1 and aspirin-triggered RvD1 exert their effects primarily through G protein–coupled receptors such as ALX/FPR2 and GPR32, regulating NF-κB, MAPK, NLRP3 inflammasome signaling, oxidative stress, and proinflammatory cytokine networks [6]. The reported ability of RvD1 to suppress neuroinflammation, support cell survival, and reduce oxidative damage in central nervous system disorders makes resolvin-family specialized pro-resolving mediators (SPMs) biologically relevant not only to epilepsy but also to inflammation-associated cerebrovascular disease, traumatic brain injury, and neurodegenerative conditions, including dementia, Parkinson’s disease, and amyotrophic lateral sclerosis [6].

The RvD1 findings in this study showed a different pattern from those of NP-1. RvD1 levels were lower within the first 3 h after an epileptic seizure and remained significantly lower than those of the control group after 1 month of seizure freedom (Table 3 and Table 4). The absence of a significant difference between the two patient time points (Table 2; Figure 1 and Figure 2) suggests that reduced RvD1 levels may reflect a more persistent biological feature rather than a brief postictal fluctuation. Their continued reduction after 1 month without seizures is consistent with incomplete recovery of resolvin-mediated pro-resolving capacity. RvD1 may therefore warrant further study as a candidate marker of persistent impairment in inflammatory resolution in epilepsy. Its potential role in supporting diagnosis during seizure-free periods, however, cannot be established from these findings alone.

The resolvin-related findings of the present study are consistent with those of Li et al., who reported reduced RvD1 and Resolvin E1 levels during the chronic course of epilepsy [7]. Li et al. found lower levels of both mediators in plasma and cerebrospinal fluid from patients with epilepsy, alongside increased levels of proinflammatory factors [7]. These findings suggest that inflammatory activation and impaired resolution of inflammation may coexist in epilepsy [7]. In the present study, RvD1 levels remained low after 1 month of seizure freedom, supporting the possibility that reduced pro-resolving capacity is not confined to the acute postictal period and may be associated with a more persistent inflammatory state. The sustained reduction in RvD1 suggests that impaired anti-inflammatory resolution may accompany chronic inflammatory activity in epilepsy (Table 3 and Table 4).

Omega-3 fatty acids serve as precursors for the synthesis of specialized pro-resolving mediators (SPMs) through docosahexaenoic acid (DHA) and eicosapentaenoic acid (EPA). They have effects on neuroinflammation, oxidative stress, synaptic function, and cell membrane dynamics [11]. DHA influences neuronal membrane fluidity, synaptic function, and neurotransmitter release, whereas EPA may limit the production of inflammatory eicosanoids and support vascular endothelial function. These properties make the omega-3 pathway relevant to epilepsy and neurodevelopmental disorders [11]. Because omega-3 fatty acids affect several interconnected processes, the broader network of lipid mediators warrants consideration in epilepsy [11]. Examining NP-1 and RvD1 together in the present study therefore allows two components of the omega-3-derived pro-resolving pathway to be assessed at the same clinical time points.

One of the most notable features of this study is the comparison of measurements obtained from the same patients within the first 3 h after a seizure and after 1 month of seizure freedom. Single-time-point biomarker measurements in epilepsy may have limited ability to distinguish acute postictal changes from features of the underlying condition. Assessing two time points in the same patients allowed us to compare the temporal patterns of NP-1 and RvD1. NP-1 was most markedly reduced in the early postictal period and showed partial recovery after 1 month without seizures, whereas RvD1 remained low at both time points. These distinct patterns suggest that biomarker assessment in epilepsy should consider changes over time, in addition to whether a measured level is high or low (Table 2; Figure 1 and Figure 2). The timing of the postictal inflammatory response is critical to this interpretation. Experimental studies of epileptogenesis have reported an early and pronounced proinflammatory cytokine response, whereas pro-resolving mechanisms appear to be activated later and may remain insufficient [5]. This delay suggests impaired progression of the inflammatory response toward resolution in epileptic tissue [5]. In the present study, the low NP-1 and RvD1 levels observed soon after a seizure are consistent with an inadequate or potentially depleted early pro-resolving response. The persistence of low RvD1 levels during the seizure-free period further suggests that this pattern may extend beyond a delay in the acute response and reflect a more sustained disturbance in pro-resolving pathways.

Disruption of blood–brain barrier integrity may increase interactions between peripheral inflammatory mediators and the central nervous system, thereby contributing to epileptogenesis [1]. The interplay between systemic and central inflammation also suggests that lipid mediators measured in peripheral blood may have clinical relevance in epilepsy [1]. Reports of reduced resolvin levels in both plasma and cerebrospinal fluid during the chronic course of epilepsy suggest that peripheral measurements may partly reflect impaired central pro-resolving activity, although they cannot fully capture it [7]. Accordingly, the serum NP-1 and RvD1 changes observed in the present study may be considered in the context of the systemic–central inflammatory axis associated with epilepsy. Nevertheless, peripheral concentrations should not be assumed to directly represent changes within central nervous system tissue [1]. The clinical relevance of these findings lies in the potential value of pro-resolving mediators in epilepsy as biomarkers and therapeutic targets, in addition to their role in understanding disease mechanisms. Unlike conventional anti-inflammatory treatments that broadly suppress immune activity, SPM-based approaches aim to restore the physiological processes that resolve inflammation [4]. In this context, resolution pharmacology offers a potential treatment framework for central nervous system disorders characterized by chronic neuroinflammation [4]. The reduction in seizure frequency and seizure burden observed with protectin D1 in an experimental epilepsy model provides preclinical support for this approach [5]. Reports of reduced RvD1 and Resolvin E1 levels in patients with epilepsy provide a rationale for further clinical research on these mediators [7].

Nevertheless, the findings of this study do not establish causality or directly support a treatment recommendation. Reduced NP-1 and RvD1 levels may be consequences of seizures or the underlying epileptic condition, or they may reflect a predisposition to impaired inflammatory resolution. Biomarker studies with limited sampling time points cannot reliably distinguish between these possibilities. Larger longitudinal studies are therefore needed, with measurements during seizures, in the postictal period, and during sustained seizure freedom. Such studies should assess proinflammatory cytokines, oxidative stress markers, omega-3 fatty acid profiles, and clinical measures of seizure severity alongside NP-1 and RvD1. Combining peripheral measurements with cerebrospinal fluid analyses or neuroimaging markers of inflammation may also clarify how closely these biomarkers reflect central nervous system processes.

Antiseizure medication (ASM) use is another important consideration when interpreting these findings. Approximately 92% of the patients in this study were receiving ASMs, with levetiracetam being the most commonly used drug (Table 1). ASMs may have direct or indirect effects on inflammation, oxidative stress, lipid metabolism, and immune responses [12]. Differences in NP-1 and RvD1 levels may therefore reflect treatment exposure, the number of medications used, seizure type and frequency, or disease duration, as well as epilepsy-related biology. Comparing the postictal and seizure-free measurements within the same patients helps reduce the influence of stable differences between individuals, although it does not eliminate the effects of treatment or other factors that may change over time.

When patients were grouped by the number of antiseizure medications (ASMs) used, NP-1 and RvD1 levels did not differ significantly between those receiving 1–2 ASMs and those receiving 3–4 ASMs, either within the first 3 h after a seizure or after 1 month of seizure freedom (all *p* > 0.05; effect sizes r < 0.10, indicating negligible or very small effects) (Table 8; Figure 7). These findings provide no evidence of an association between the number of ASMs used and circulating NP-1 or RvD1 levels in this sample. They do not, however, exclude effects of specific drugs or establish that the observed biomarker patterns are independent of treatment. Likewise, ASM count alone does not establish drug resistance, so these comparisons cannot determine whether either marker can identify or predict drug-resistant epilepsy.

Larger studies that separately assess newly diagnosed, treatment-naive patients, patients with clinically defined drug-resistant epilepsy, and patients with a good treatment response may clarify how pro-resolving mediators relate to epilepsy phenotypes and treatment exposure.

The heterogeneity of epilepsy poses a challenge for biomarker research, particularly when findings are examined by seizure type. In the present study, NP-1 and RvD1 levels did not differ significantly across seizure subtypes (Table 7; Figure 6). Focal motor, generalized motor, focal nonmotor, and generalized nonmotor seizures may involve different network dynamics and produce different inflammatory and systemic responses. Although these subgroups were assessed, their limited sample sizes preclude firm conclusions about seizure type–specific biomarker patterns. The differences observed in the overall epilepsy group raise the possibility that altered pro-resolving activity extends across seizure types, but this requires confirmation in larger subtype-specific cohorts. This interpretation is consistent with reports of neuroinflammation across several epilepsy phenotypes, including temporal lobe epilepsy, frontal lobe epilepsy, and focal cortical dysplasia [1].

The choice of two sampling points—within the first 3 h after an epileptic seizure and after 1 month of seizure freedom—has both strengths and limitations. These time points were selected to compare acute postictal variation in inflammatory and pro-resolving biomarkers with levels measured during a more stable period, when the immediate effects of the seizure were less likely to predominate. Sampling in the early postictal period is therefore relevant to capturing seizure-associated immune and neuroinflammatory responses [12,13,14]. The first 3 h were chosen specifically to represent this early biological response. Human studies have reported increases in immune markers after a single epileptic seizure, with changes temporally associated with the event [13]. Studies of childhood febrile seizures and acute encephalopathy have likewise described dynamic changes in cytokines during the first hours after a seizure, with some markers tending to decline within 12–24 h [14].

However, the first 3 h after a seizure may not capture the peak of the pro-resolving response, as experimental models of epileptogenesis indicate that pro-resolving pathways can be activated later than inflammatory cytokine pathways [5]. Frigerio et al. reported increased IL-1β and TNF-α transcript levels in the mouse hippocampus from 2 to 72 h after status epilepticus, whereas enzymes involved in the synthesis of pro-resolving mediators, including 5-lipoxygenase and 15-lipoxygenase, and certain pro-resolving receptors became more prominent later [5]. These findings suggest that the first hours after a seizure primarily capture acute inflammatory activation, whereas the pro-resolving response may emerge later or remain insufficient. Thus, sampling within 3 h was appropriate for examining the early behavior of NP-1 and RvD1 after a seizure, but this window cannot characterize the full time course of the pro-resolving response. The second sample, obtained after 1 month of seizure freedom, was intended to assess a short-term seizure-free period beyond the immediate postictal phase. Inflammatory markers in epilepsy may change during the interictal period, and chronic inflammatory activity may persist in some patient groups [7,13,15]. A second measurement during seizure freedom may therefore help distinguish acute postictal changes from more sustained biological differences. Although 1 month is a practical interval for short-term follow-up, definitions of the interictal period vary across studies: some use a minimum of 72 h after the last seizure, while others report declining cytokine levels during seizure-free periods [16,17].

However, these studies do not define standardized sampling windows specifically for NP-1 or RvD1. One month of seizure freedom nevertheless provides a comparison point beyond the immune fluctuations reported during the first 24–72 h after a seizure, when the immediate postictal biochemical changes might be expected to have largely subsided. Thus, there is a rationale for sampling within 3 h after a seizure and again after 1 month of seizure freedom. The lack of consensus on the timing of inflammatory and pro-resolving responses in epilepsy, together with the absence of established sampling windows for NP-1 and RvD1, remains a limitation of the present study.

The contribution of this study lies in measuring NP-1 and RvD1 in the same patients at two clinically distinct time points: early after a seizure and after a period of seizure freedom (Table 2; Figure 1, Figure 2 and Figure 3). Comparing their temporal patterns offers a view of how markers associated with inflammatory resolution may change over the course of epilepsy. Although the role of specialized pro-resolving mediators in central nervous system disorders is receiving increasing attention, human studies examining how specific pro-resolving molecules vary across clinical phases of epilepsy remain limited [4].

Recent clinical evidence of reduced RvD1 and Resolvin E1 levels during the chronic course of epilepsy indicates that human research on pro-resolving mediators is still developing [7]. The present study extends this work by examining NP-1 and RvD1 at two distinct time points: early after a seizure and after 1 month of seizure freedom. These measurements provide a clinical basis for investigating whether markers associated with inflammatory resolution show different patterns in the postictal and seizure-free periods.

In conclusion, the principal finding of this study is that the pro-resolving lipid mediator profile appears altered in patients with epilepsy both early after a seizure and after a period of seizure freedom. NP-1 levels were particularly low in the early postictal period and showed partial recovery after 1 month without seizures. This pattern suggests that NP-1 may be a state-dependent marker sensitive to seizure-associated changes in the pro-resolving response. RvD1 levels, in contrast, remained lower than those of the control group both early after a seizure and after 1 month of seizure freedom. This pattern suggests that reduced RvD1 may represent a more persistent, trait-like feature of epilepsy, although longer follow-up is needed to establish its stability. Considered together, the distinct NP-1 and Resolvin D1 patterns are consistent with altered inflammatory resolution in epilepsy, alongside the inflammatory activation described in previous research. Both molecules merit further investigation as candidate biomarkers of seizure-associated change and seizure-free status. Their relevance as treatment targets remains to be established.

## Figures and Tables

**Table 1:** Demographic and Clinical Characteristics.

Variable	*n* (%)/Value
Sex, *n* (%)	
Female	30 (62.5)
Male	18 (37.5)
Age (years)	
Mean ± SD	41.88 ± 14.82
Median [IQR]	38 [31–50]
Min–Max	20–87
Number of medications, *n* (%)	
0 (newly diagnosed/untreated)	4 (8.3)
1	18 (37.5)
2	13 (27.1)
3	10 (20.8)
4	3 (6.2)
Antiseizure medications used *, *n* (%)	
Levetiracetam	28 (58.3)
Valproic acid	13 (27.1)
Lamotrigine	9 (18.8)
Topiramate	8 (16.7)
Lacosamide	7 (14.6)
Clonazepam	7 (14.6)
Phenytoin	5 (10.4)
Clobazam	3 (6.2)
Zonisamide	2 (4.2)
Phenobarbital	1 (2.1)
Primidone	1 (2.1)
Carbamazepine	1 (2.1)
Pregabalin	1 (2.1)
Seizure type, *n* (%)	
Generalized motor	29 (60.4)
Focal motor	9 (18.8)
Generalized nonmotor	7 (14.6)
Focal nonmotor	3 (6.2)

* Patients could receive more than one medication, so percentages exceed 100%. SD: standard deviation; IQR: interquartile range (25th–75th percentiles). Sample size: *n* = 48. Medication names are written in full in the table.

**Table 2:** Comparison of NP-1 and RvD1 Levels During the EN and ER Periods in Patients with Epilepsy (*n* = 48).

Marker	EN (Postictal)	ER (Seizure-Free Period)	Z	*p*	r
NP-1 (ng/mL), median [IQR]	50.01 [3.58–98.62]	67.78 [59.81–121.81]	−5.333	<0.001	0.770
NP-1, mean ± SD	58.79 ± 53.91	91.13 ± 47.21	–	–	–
RvD1 (pg/mL), median [IQR]	326.67 [207.77–899.95]	441.47 [223.67–710.81]	−1.487	0.139	0.215
RvD1, mean ± SD	525.56 ± 404.77	531.52 ± 369.13	–	–	–

The Wilcoxon signed-rank test was applied to paired samples. EN: early postictal period, within 3 h after the index seizure; ER: seizure-free period, after at least 1 month without seizures; NP-1: neuroprotectin D1; RvD1: resolvin D1; IQR: interquartile range; SD: standard deviation; Z: standardized test statistic; r: effect size (|Z|/√N; *n* = 48 paired observations). The significance threshold was *p* < 0.05.

**Table 3:** Comparison of NP-1 and RvD1 Levels Between the Control and EN Groups.

Marker	Control (*n* = 50)	EN (*n* = 48)	U	Z	*p*	r
NP-1 (ng/mL), median [IQR]	118.02 [84.02–167.51]	50.01 [3.58–98.62]	1849	4.612	<0.001	0.466
NP-1, mean ± SD	118.31 ± 53.43	58.79 ± 53.91	–	–	–	–
RvD1 (pg/mL), median [IQR]	1124.37 [742.46–1258.38]	326.67 [207.77–899.95]	1875	4.797	<0.001	0.485
RvD1, mean ± SD	953.91 ± 421.49	525.56 ± 404.77	–	–	–	–

The Mann–Whitney U test was applied. EN: early postictal period (within 3 h); NP-1: neuroprotectin D1; RvD1: resolvin D1; IQR: interquartile range; SD: standard deviation; U and Z: test statistics; r: effect size (|Z|/√N; *n* = 98). *p*-values are unadjusted and exploratory.

**Table 4:** Comparison of NP-1 and RvD1 Levels Between the Control and ER Groups.

Marker	Control (*n* = 50)	ER (*n* = 48)	U	Z	*p*	r
NP-1 (ng/mL), median [IQR]	118.02 [84.02–167.51]	67.78 [59.81–121.81]	1576	2.672	0.008	0.270
NP-1, mean ± SD	118.31 ± 53.43	91.13 ± 47.21	–	–	–	–
RvD1 (pg/mL), median [IQR]	1124.37 [742.46–1258.38]	441.47 [223.67–710.81]	1858	4.680	<0.001	0.473
RvD1, mean ± SD	953.91 ± 421.49	531.52 ± 369.13	–	–	–	–

The Mann–Whitney U test was applied. ER: seizure-free period (at least 1 month); NP-1: neuroprotectin D1; RvD1: resolvin D1; IQR: interquartile range; SD: standard deviation; U and Z: test statistics; r: effect size (|Z|/√N; *n* = 98). *p*-values are unadjusted and exploratory.

**Table 5:** Comparison of Changes in NP-1 and RvD1 Relative to Controls in the EN Group.

Marker	%Δ Median [IQR]	|%Δ| Median [IQR]	T	Z	*p*	r
NP-1 (*n* = 48)	−57.63 [−96.97; −16.44]	57.63 [39.47; 96.97]	467	1.241	0.219	0.179
RvD1 (*n* = 48)	−70.95 [−81.52; −19.96]	70.95 [19.96; 81.52]	–	–	–	–

The comparison used absolute percentage differences (|%Δ|) and the paired Wilcoxon test. EN: early postictal period (within 3 h); NP-1: neuroprotectin D1; RvD1: resolvin D1; IQR: interquartile range; %Δ: percentage difference from the corresponding control median; T and Z: test statistics; r: effect size (*n* = 48).

**Table 6:** Comparison of Changes in NP-1 and RvD1 Relative to Controls in the ER Group.

Marker	%Δ Median [IQR]	|%Δ| Median [IQR]	T	Z	*p*	r
NP-1 (*n* = 48)	−42.57 [−49.33; +3.21]	42.84 [40.42; 49.33]	276	−3.200	0.001	0.462
RvD1 (*n* = 48)	−60.74 [−80.11; −36.78]	60.74 [36.78; 80.11]	–	–	–	–

The comparison used absolute percentage differences (|%Δ|) and the paired Wilcoxon test. ER: seizure-free period (at least 1 month); NP-1: neuroprotectin D1; RvD1: resolvin D1; IQR: interquartile range; %Δ: percentage difference from the corresponding control median; T and Z: test statistics; r: effect size (*n* = 48).

**Table 7:** NP-1 and RvD1 Levels During EN and ER by Seizure Subtype.

Marker/Period	Gen. Motor (*n* = 29)	Focal Motor (*n* = 9)	Gen. Nonmotor (*n* = 7)	Focal Nonmotor (*n* = 3)	H	*p*
NP-1, EN period	63.33 [4.95–108.72]	3.65 [3.26–42.83]	52.18 [50.01–82.96]	17.38 [10.12–55.56]	3.25	0.354
NP-1, ER period	80.41 [59.75–167.15]	65.80 [61.63–112.65]	65.22 [60.86–74.35]	66.81 [35.30–90.95]	1.63	0.653
RvD1, EN period	367.46 [214.68–1020.03]	220.28 [167.17–354.02]	356.74 [246.90–685.97]	278.12 [251.23–584.72]	2.26	0.521
RvD1, ER period	447.36 [216.91–1003.10]	435.58 [228.02–667.63]	326.90 [292.81–466.12]	501.64 [355.76–663.66]	0.23	0.973

Kruskal–Wallis H test (df = 3), applied separately within each time point. Values are medians [IQR]. EN: early postictal period (within 3 h); ER: seizure-free period (at least 1 month); NP-1: neuroprotectin D1; RvD1: resolvin D1; H: test statistic. NP-1 is reported in ng/mL, and RvD1 in pg/mL.

**Table 8:** Comparison of NP-1 and RvD1 Levels by Number of Antiseizure Medications Used.

Period/Marker	1–2 Medications (*n* = 31), Median [IQR]	3–4 Medications (*n* = 13), Median [IQR]	U	Z	*p*	r
EN (postictal)						
NP-1 (ng/mL)	50.05 [3.59–105.02]	49.97 [3.65–79.15]	226	0.630	0.537	0.095
RvD1 (pg/mL)	299.31 [213.36–972.42]	356.74 [204.55–618.64]	223	0.553	0.589	0.083
ER (after 1 month without seizures)						
NP-1 (ng/mL)	68.76 [59.53–145.50]	72.17 [61.57–87.38]	224	0.579	0.571	0.087
RvD1 (pg/mL)	435.58 [229.23–843.93]	468.80 [214.94–611.09]	213	0.296	0.777	0.045

The Mann–Whitney U test was applied. Four untreated patients were excluded. EN: early postictal period (within 3 h); ER: seizure-free period (at least 1 month); NP-1: neuroprotectin D1; RvD1: resolvin D1; IQR: interquartile range; U and Z: test statistics; r: effect size (|Z|/√N; *n* = 44).

## Data Availability

Upon request, the necessary data may be shared with the editor, while ensuring the protection of the participants’ personal data.
